# A novel machine learning prediction model for metastasis in breast cancer

**DOI:** 10.1002/cnr2.2006

**Published:** 2024-02-29

**Authors:** Huan Li, Ren‐Bin Liu, Chen‐meng Long, Yuan Teng, Yu Liu

**Affiliations:** ^1^ Department of Thyroid and Breast Surgery Third Affiliated Hospital of Sun Yat‐sen University Guangzhou China; ^2^ Department of Breast Surgery Liuzhou Women and Children's Medical Center Liuzhou China; ^3^ Department of Breast Surgery Guangzhou Women and Children's Medical Center Guangzhou China

**Keywords:** breast cancer, metastasis, predictive model, random survival forest, recursive feature elimination

## Abstract

**Background:**

Breast cancer (BC) metastasis is the common cause of high mortality. Conventional prognostic criteria cannot accurately predict the BC metastasis risk. The machine learning technologies can overcome the disadvantage of conventional models.

**Aim:**

We developed a model to predict BC metastasis using the random survival forest (RSF) method.

**Methods:**

Based on demographic data and routine clinical data, we used RSF‐recursive feature elimination to identify the predictive variables and developed a model to predict metastasis using RSF method. The area under the receiver operating characteristic curve (AUROC) and Kaplan–Meier survival (KM) analyses were plotted to validate the predictive effect when C‐index was plotted to assess the discrimination and Brier scores was plotted to assess the calibration of the predictive model.

**Results:**

We developed a metastasis prediction model comprising three variables (pathological stage, aspartate aminotransferase, and neutrophil count) selected by RSF‐recursive feature elimination. The model was reliable and stable when assessed by the AUROC (0.932 in training set and 0.905 in validation set) and KM survival analyses (*p* < .0001). The C‐indexes (0.959) and Brier score (0.097) also validated the good predictive ability of this model.

**Conclusions:**

This model relies on routine data and examination indicators in real‐time clinical practice and exhibits an accurate prediction performance without increasing the cost for patients. Using this model, clinicians can facilitate risk communication and provide precise and efficient individualized therapy to patients with breast cancer.

## 1 INTRODUCTION

Breast cancer (BC) is the most common malignant tumor and the primary cause of tumor mortality in women globally.^1^ The morbidity has also been increasing annually in patients with BC. The new cases of female BC are estimated to be 2.3 million according to GLOBOCAN 2020; thus, BC has surpassed lung cancer and become the most common cancer in women.^1^ Owing to dietary regimens, lifestyle changes, and the natural environment, the morbidity and mortality rates of BC have increased in China recent years.^2^, ^3^

Most of breast cancer‐related deaths are associated with metastasis that over 90% are attributed to metastasis‐related complications.^4^ Metastatic BC remains incurable despite improvements in early detection and advances in treatment because metastatic BC is refractory to almost all current treatments and most of treatments are not curative but just merely palliative.^4^, ^5^ On one hand, identification of BC metastasis risk could inform approaches to early detection and prevention by additional interventions. On another hand it is critical to accurately predict metastasis for precision medicine and individualized therapy, thus avoiding the need for toxic and costly therapies. However, inappropriate screening examinations or the overuse of diagnostic tests has increased healthcare costs. Conventional predictors for BC metastases, such as histological grade and lymph node status, are limited in their ability to accurately predict metastases corresponding to clinical symptoms.^6^, ^7^, ^8^ Therefore, conventional prognostic criteria cannot predict the metastasis risk accurately in patients with BC. Consequently, many patients unnecessarily receive cytotoxic chemotherapy.^8^ Accurate prediction of BC metastasis risks could help to reduce the public health and social burdens of breast cancer. As the gene technology developed, researchers have applied the integration of multiple genetic and molecular markers to develop newer models to predict the prognosis of breast cancer patients. Some multigene expression assays, such as the 21‐gene recurrence score assay and the 70‐gene expression profile, have been developed to identify patients with a high risk of developing to recurrence or metastasis and who would thus benefit from chemotherapy.^8^, ^9^, ^10^ However, gene detection requires cutting‐edge technology, making it expensive. Furthermore, its utility has only been established in a certain patient subset^8^, ^11^, ^12^, ^13^; thus, it is not widely used.

Recently, researchers have developed several prediction models based on pathological and clinical factors.^14^, ^15^, ^16^, ^17^ PREDICT and Adjuvant! Online are commonly applied and can calculate individualized survival probabilities based on integrating clinical variables (tumor size, nodal involvement, histologic grade, hormone receptor status, and age) into a multivariable statistical analysis relying on agnostic survival analysis statistical tools, such as Cox regression.^14^, ^15^, ^16^, ^17^ Cox regression is generally employed to identify predictors but involves restrictive assumptions, such as proportionality of hazards and linearity,^18^ which may introduce bias into the prognostic analysis of BC patients during long‐term follow‐up and hinder the identification of prognostic markers.^19^, ^20^ So, a simple and accurate predictive model with high clinical applicability and generalizability is needed to predict BC metastasis urgently.

The machine learning methods can construct predictive models that can evaluate numerous variables efficiently, overcoming the disadvantage of conventional models. Random survival forest (RSF), developed from random forest and survival analysis, is a machine‐learning method^21^ that has no restrictions on the data distribution, making it a non‐parametric method and can be applied to analyze data with a significantly larger number of variables than the sample size. Moreover, this method can avoid potential overfitting and collinearity effectively.^22^, ^23^, ^24^ Further, there are no special requirements for the data type or the association between outcomes and predictive variables, and it is not constrained by logarithmic linear assumption or proportional risk assumption.^21^, ^25^ We employed the RSF method based on baseline clinical parameters, including general information of patients, pathological examinations, and blood tests to develop a new predictive model for BC metastasis occurrence.

## 2 PATIENTS AND METHODS

### 2.1 Patients and study design

**FIGURE 1:**
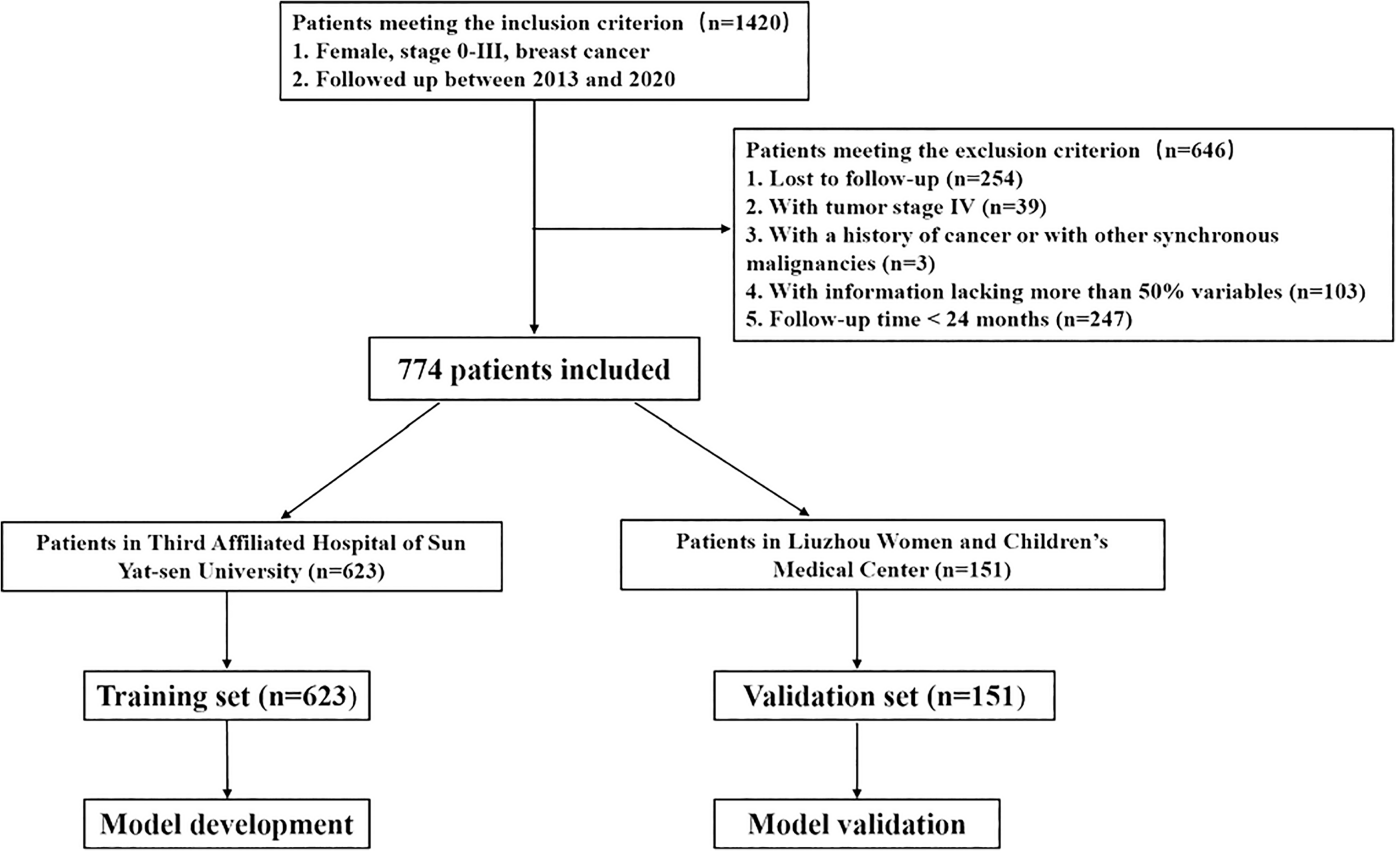
Flowchart of study design and patient selection.

We retrospectively investigated the medical records of BC patients two independent institutions from January 2013 to December 2020. Patients with stage 0 to III primary BC were enrolled and all enrolled patients accepted primary BC treatment. Patients with stage IV breast cancer, with other synchronous malignancies, with other cancer history, with incomplete information (lacking >50% parameters) or lost to follow‐up were excluded. Patients from the third affiliated hospital of Sun Yat‐sen University were assign to the training set to develop the model, and patients from Liuzhou women and children's medical center were assign to the validation set validate the model. Figure 1 presented the flowchart of the study design and patient selection.

### 2.2 Statistical analyses

The baseline demographic and clinical characteristics of patients were presented as percentages or means with standard deviations. Continuous data were evaluated using the Student's *t*‐test or the Mann–Whitney *U* test, and categorical data were evaluated using the Chi‐squared test. All statistical analyses were performed using R software (R Foundation for Statistical Computing, Vienna, Austria). All analyses were two‐tailed, and differences were statistically significant at *p* < .05.

### 2.3 Data preprocessing

Missing data in the training and validation sets were interpolated by the “mice” package in R (predictive mean matching [PMM]).

### 2.4 Predictor selection

Based on the clinical date, random forest‐recursive feature elimination (RF‐RFE) (run by the “caret” package in R) was applied to select the best variable set (a positive variable importance [VIMP] value calculated by RF‐RFE indicates that one variable improves predictive accuracy, while a negative value indicates an adverse effect in the prediction).^26^

### 2.5 Model development and evaluation

The RSF method was applied to develop a model to predict BC metastasis risk. To evaluate the accuracy of the model, we took the root mean square error (RMSE) that occurred mathematically between the test and predicted values. The higher the prediction accuracy, the lower the RMSE.^27^ All pairs of mtry and ntree were developed by a grid search employing 10‐fold cross‐validation, and those with the best concordance index (C‐index) were determined as optimized parameters. We used the C‐index to assess the discrimination of the predictive model (0.5–0.7 represents weak discrimination power, 0.7–0.9 represents moderate discrimination power, and >0.9 represents strong discrimination power).^28^ We used the Brier scores to evaluate the calibration of the model. The Brier score measures the calibration of the model by taking the mean squared error between the predicted probabilities and the observed outcomes. It ranges from 0 to 1, a lower score indicating higher accuracy.^29^, ^30^ Brier scores <0.25 showed relatively good calibration from automated modeling.^29^, ^30^ A receiver operating characteristic (ROC) curve and Kaplan–Meier (KM) survival analysis were applied to assess the precision of the predictive model. BC metastasis is the end‐point event.

## 3 RESULTS

We enrolled 774 patients, 623 patients were included in the training and 151 patients were included in validation sets for model development and model validation, respectively. Forty‐one variables were included and 22 variables needed interpolation in training set and 11 variables needed interpolation in validation set. All missing data with missing rate less than 20%. Baseline characteristic of the training and validation sets is present in Table 1.

**TABLE 1:** Basic information of the training and validation sets.

	Total set	Training set	Validation set	*p* value
Total (*n*)	774	623	151	‐
Female sex (*n*)	774	623	151	‐
Age (years)	49.76 ± 10.83	50.50 ± 10.84	46.74 ± 10.27	.001
≥60 (*n*)	155 (20.03%)	138 (22.15%)	17 (11.26%)	.011
<60 (*n*)	619 (79.97%)	485 (77.85%)	134 (88.74%)	
≥35 (*n*)	713 (92.12%)	577 (92.62%)	136 (90.07%)	.569
<35 (*n*)	61 (7.88%)	46 (7.38%)	15 (9.93%)	
BMI (kg/m^2^)	23.19 ± 3.18	23.69 ± 3.36	21.49 ± 1.54	<.001
ALT (U/L)	20.24 ± 12.94	19.52 ± 13.84	23.23 ± 7.59	<.001
AST (U/L)	21.0 ± 10.02	21.34 ± 11.07	19.62 ± 2.59	.167
TBIL (μmol/L)	11.12 ± 4.82	10.92 ± 4.34	11.96 ± 6.34	.312
DBIL (μmol/L)	3.31 ± 1.45	3.18 ± 1.46	3.81 ± 1.26	<.001
GGT (U/L)	25.40 ± 17.80	26.81 ± 8.62	25.03 ± 19.46	<.001
ALP (U/L)	64.84 ± 20.94	65.14 ± 22.63	63.66 ± 12.14	.738
ALB (g/L)	42.63 ± 4.37	42.59 ± 3.58	42.80 ± 6.72	.875
GLB (g/L)	27.30 ± 4.21	27.15 ± 3.99	27.88 ± 5.02	.169
A/G	1.60 ± 0.28	1.60 ± 0.25	1.58 ± 0.37	.821
Cr (μmol/L)	65.13 ± 17.03	59.88 ± 12.19	86.71 ± 17.15	<.001
GLU	5.40 ± 1.73	5.47 ± 1.41	5.13 ± 2.67	<.001
UA	313.78 ± 80.62	315.80 ± 89.10	305.96 ± 29.50	.409
CHOL	4.98 ± 0.94	4.94 ± 1.04	5.12 ± 0.36	.112
TRIG	1.24 ± 0.88	1.35 ± 0.95	0.81 ± 0.34	<.001
HDL	1.36 ± 0.45	1.30 ± 0.32	1.59 ± 0.73	<.001
LDL	3.06 ± 0.87	3.11 ± 0.90	2.88 ± 0.76	.017
ApoA	1.47 ± 0.22	1.43 ± 0.22	1.62 ± 0.09	<.001
ApoB	1.01 ± 0.27	1.03 ± 0.30	0.94 ± 0.07	.005
Lpa	198.07 ± 217.58	210.55 ± 241.54	149.30 ± 38.15	.009
WBC (×10^9^/L)	5.88 ± 1.81	6.28 ± 1.65	4.25 ± 1.46	<.001
NEUT (×10^9^/L)	3.69 ± 1.40	3.91 ± 1.42	2.75 ± 0.81	<.001
LYMPH (×10^9^/L)	1.77 ± 0.58	1.82 ± 0.6	1.53 ± 0.41	<.001
RBC (×10^12^/L)	4.42 ± 0.52	4.43 ± 0.55	4.37 ± 0.35	.449
HCT	0.37 ± 0.04	0.38 ± 0.04	0.35 ± 0.02	<.001
Hb (g/L)	124.94 ± 12.18	125.60 ± 13.25	122.22 ± 5.15	.009
PLT (×10^9^/L)	239.73 ± 65.38	253.51 ± 63.0	182.87 ± 39.08	<.001
AST/PLT	0.09 ± 0.11	0.09 ± 0.12	0.11 ± 0.02	.268
NLR	2.29 ± 1.27	2.39 ± 1.37	1.85 ± 0.59	<.001
PLR	146.78 ± 57.36	152.87 ± 61.37	121.68 ± 23.51	<.001
PT (s)	12.86 ± 0.71	12.95 ± 0.74	12.51 ± 0.36	<.001
INR	0.99 ± 0.36	1.00 ± 0.39	0.95 ± 0.03	.799
Follow‐up (months)	55.59 ± 25.43	60.24 ± 25.68	36.42 ± 11.77	<.001
Tumor pathology				
Tumor stage				.569
0	29 (3.75%)	27 (4.33%)	2 (1.32%)	
I	191 (24.68%)	117 (18.78%)	74 (49.01%)	
II	382 (49.35%)	330 (52.97%)	52 (34.44%)	
III	172 (22.22%)	149 (23.92%)	23 (15.23%)	
Histology				.569
Invasive ductal carcinoma	650 (83.98%)	540 (86.68%)	110 (72.85%)	
Invasive lobular carcinoma	35 (4.52%)	26 (4.17%)	9 (5.96%)	
Carcinoma in situ	51 (6.59%)	32 (5.14%)	19 (12.58%)	
Special types (inflammatory breast cancer, Paget's disease, mucinous carcinoma, malignant phyllodes tumor)	38 (4.91%)	25 (4.01%)	13 (8.61%)	
Immunohistochemistry				
ER status				.005
Negative	150 (19.38%)	135 (21.67%)	15 (9.93%)	
Positive	624 (80.62%)	488 (78.33%)	136 (90.07%)	
PR				.027
Negative	188 (24.29%)	164 (26.32%)	24 (15.89%)	
Positive	586 (75.71%)	459 (73.68%)	127 (84.11%)	
HER2 status				.001
Negative	543 (70.16%)	418 (67.09%)	125 (82.78%)	
Positive	231 (29.84%)	205 (32.91%)	26 (17.22%)	
Ki‐67				.028
<14%	257 (33.20%)	193 (30.98%)	64 (42.38%)	
≥15%	517 (66.80%)	430 (69.02%)	87 (57.62%)	
Axillary lymph node metastasis				.023
No	446 (57.62%)	344 (55.22%)	102 (67.55%)	
Yes	328 (42.38%)	279 (44.78%)	49 (32.45%)	
Molecular type				.023
Luminal A	203 (26.23%)	162 (26.0%)	41 (27.15%)	
Luminal B	414 (53.49%)	343 (55.06%)	71 (47.02%)	
HER2 enriched	64 (8.27%)	54 (8.67%)	10 (6.62%)	
TNBC	80 (10.34%)	51 (8.19%)	29 (19.21%)	
Metastasis				.938
No	688 (88.89%)	553 (88.76%)	135 (89.40%)	
Yes	86 (11.11%)	70 (11.24%)	16 (10.6%)	
Metastatic time (months)	53.47 ± 25.11	58.04 ± 25.45	34.74 ± 11.08	<.001

Abbreviations: A/G, albumin to globulin ratio; ALB, albumin; ALP, alkaline phosphatase; ALT, alanine aminotransferase; ApoA, apolipoprotein A; ApoB, apolipoprotein B; AST, aspartate aminotransferase; BMI, body mass index; CHOL, total cholesterol; Cr, creatinine; DBIL, direct bilirubin; ER, estrogen receptor; GGT, gamma‐glutamyl transpeptidase; GLB, globulin; GLU, glucose; Hb, hemoglobin; HCT, hematocrit; HDL, high‐density lipoprotein; HER2, human epidermal growth factor receptor 2; INR, international normalized ratio; LDL, low‐density lipoprotein; Lpa, lipoprotein a; LYMPH, lymphocyte; NEUT, neutrophil; NLR, neutrophil count to lymphocyte count ratio; PLR, platelet count to lymphocyte count ratio; PLT, platelet; PR, progesterone receptor; PT, prothrombin time; RBC, red blood cell; TBIL, total bilirubin; TNBC, triple negative breast cancer; TRIG, triglyceride; UA, uric acid; WBC, white blood cell.

**FIGURE 2:**
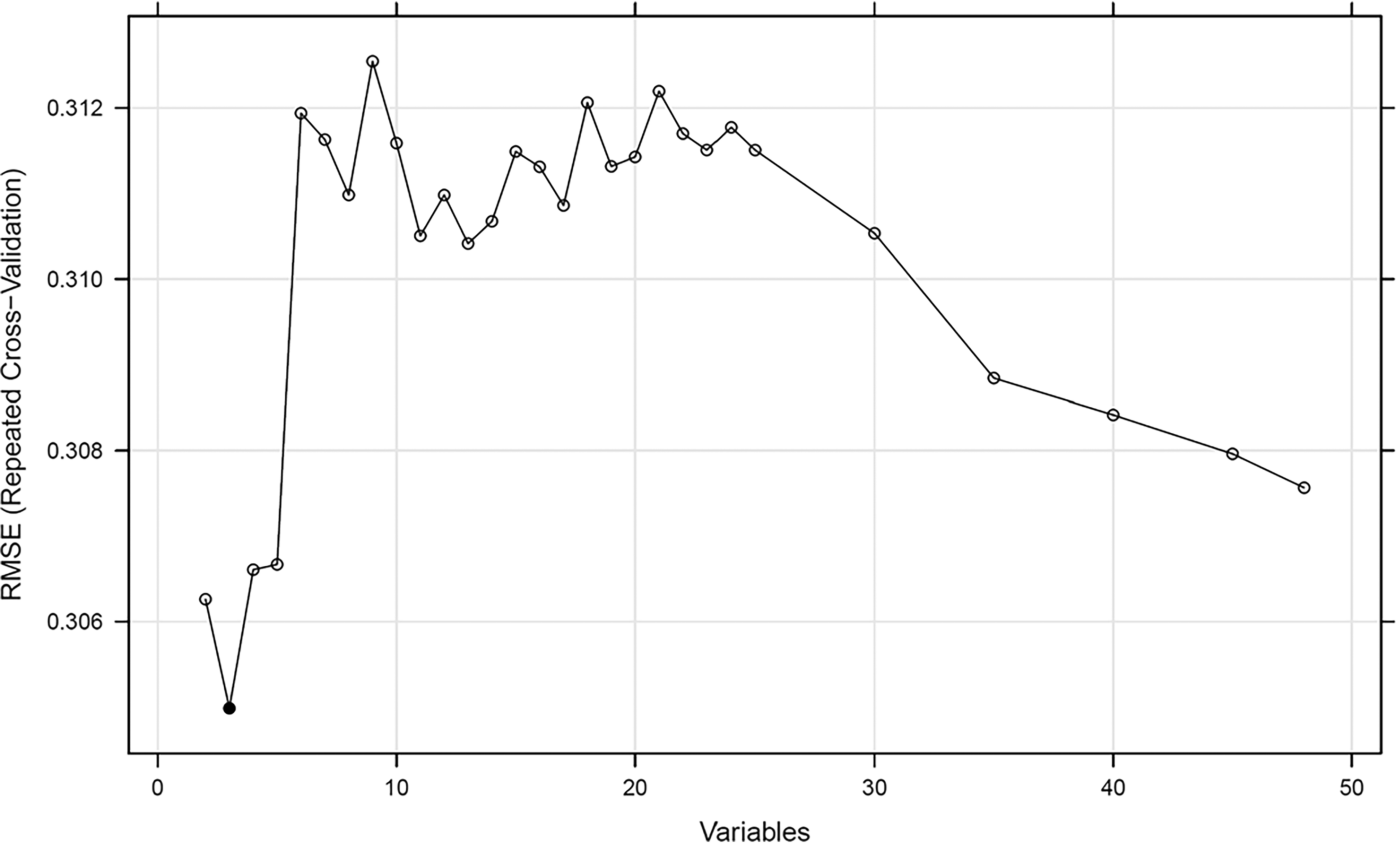
Evaluation of the number of variables in the optimal set using the root mean square error (RMSE).

The RF‐RFE run using the R “caret” package was applied to filter the most predictive set of variables, and the optimal number of variable sets was selected according to RMSE. Figure 2 shows that the RMSE value was the lowest when there were three variables; thus, the three‐variable set was the most predictive variable.

**FIGURE 3:**
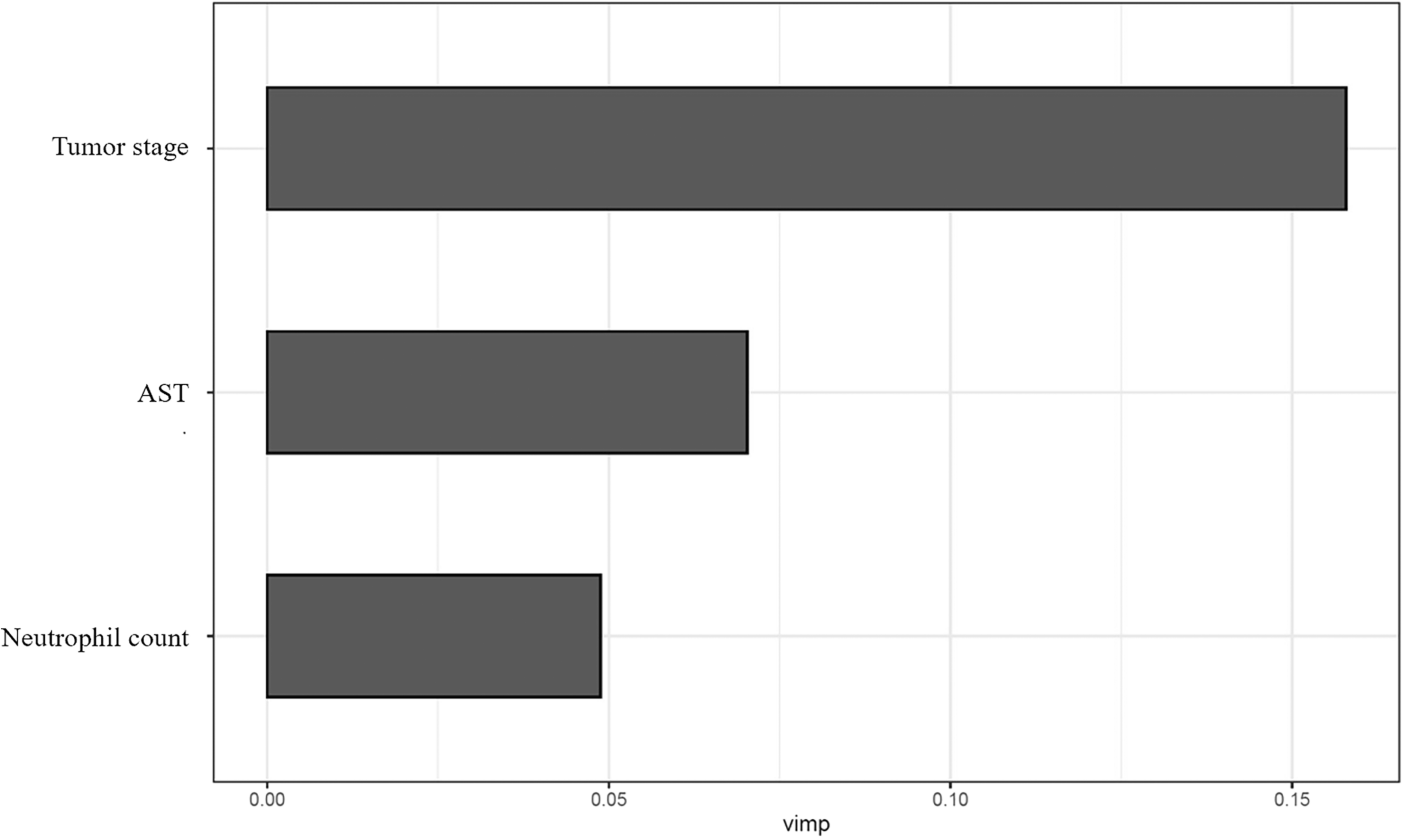
The variable importance (VIMP) values derived from random forest‐recursive feature elimination analysis.

The RF‐RFE algorithm automatically reviewed the general information of the patients, pathological examinations, and blood tests during treatment to select the most relevant features for further RSF model development. The best three variables filtered by RF‐RFE comprised pathological (TNM) stage, aspartate aminotransferase (AST), and neutrophil count. The VIMP values calculated by RF‐RFE are present in Figure 3. However, no variables from general information were selected by the RF‐RFE algorithm.

**FIGURE 4:**
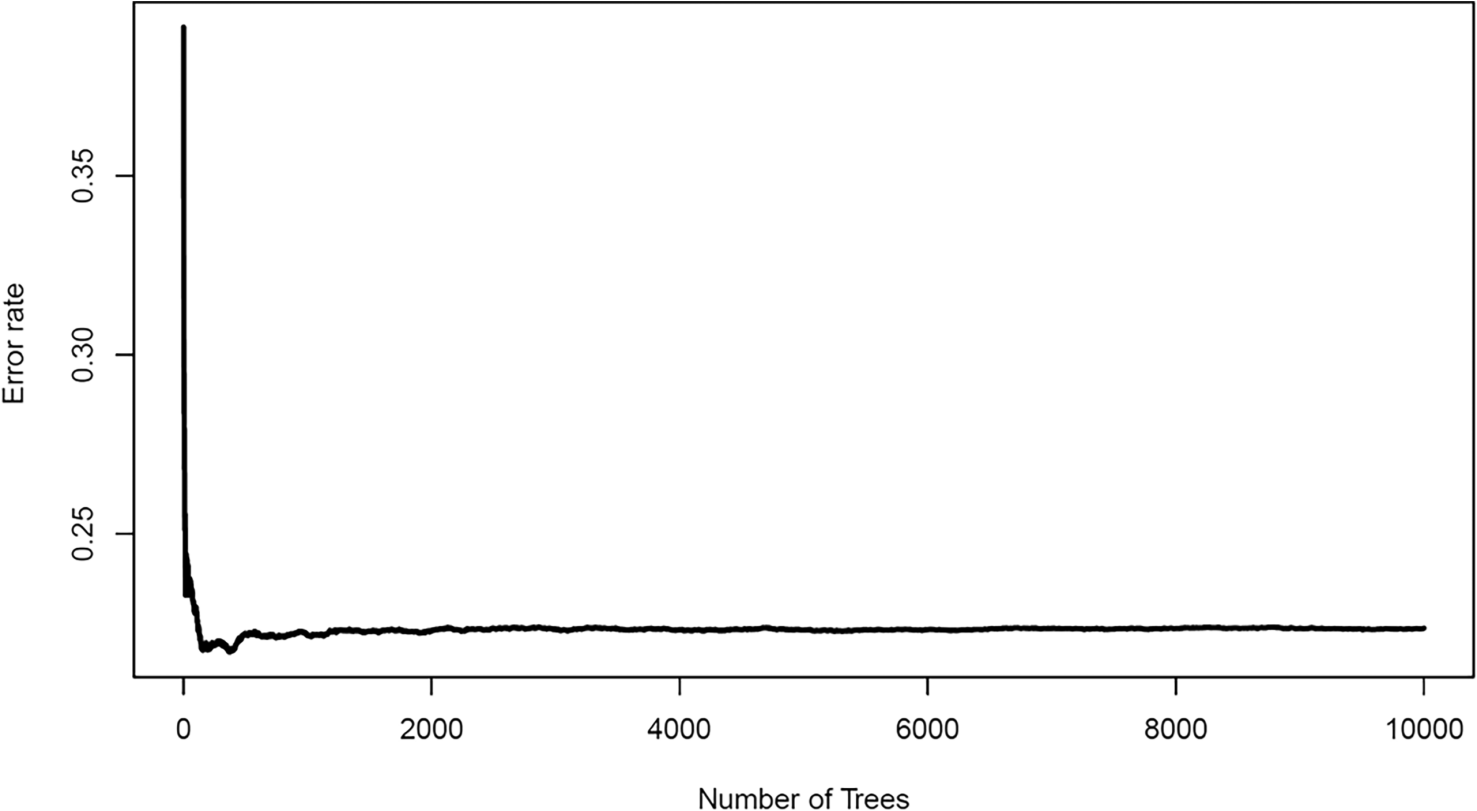
Change in the prediction error rate of metastasis risk model of breast cancer patients with tree number.

RSF with the “RandomForestSRC” package in R was applied to develop the model. The error rate of the model gradually stabilizes with the increase in the number of fixed trees (Figure 4). Between 4000 and 6000, the out‐of‐bag error rate decreases steadily, reaching close to 0.3, and when the fix trees were 10 000, the error rate is significantly stable. Thus, it is sufficient and reasonable to select 10 000 trees (ntree = 10 000), and the best predictive variables (ntree = 10 000, mtry = 4) were chosen for the development of the RSF predictive model. And then RSF‐based scores of individual were calculated. The C‐index was 0.959 (95% confidence interval [CI], 0.918–0.999) when the Brier score was 0.113.

**FIGURE 5:**
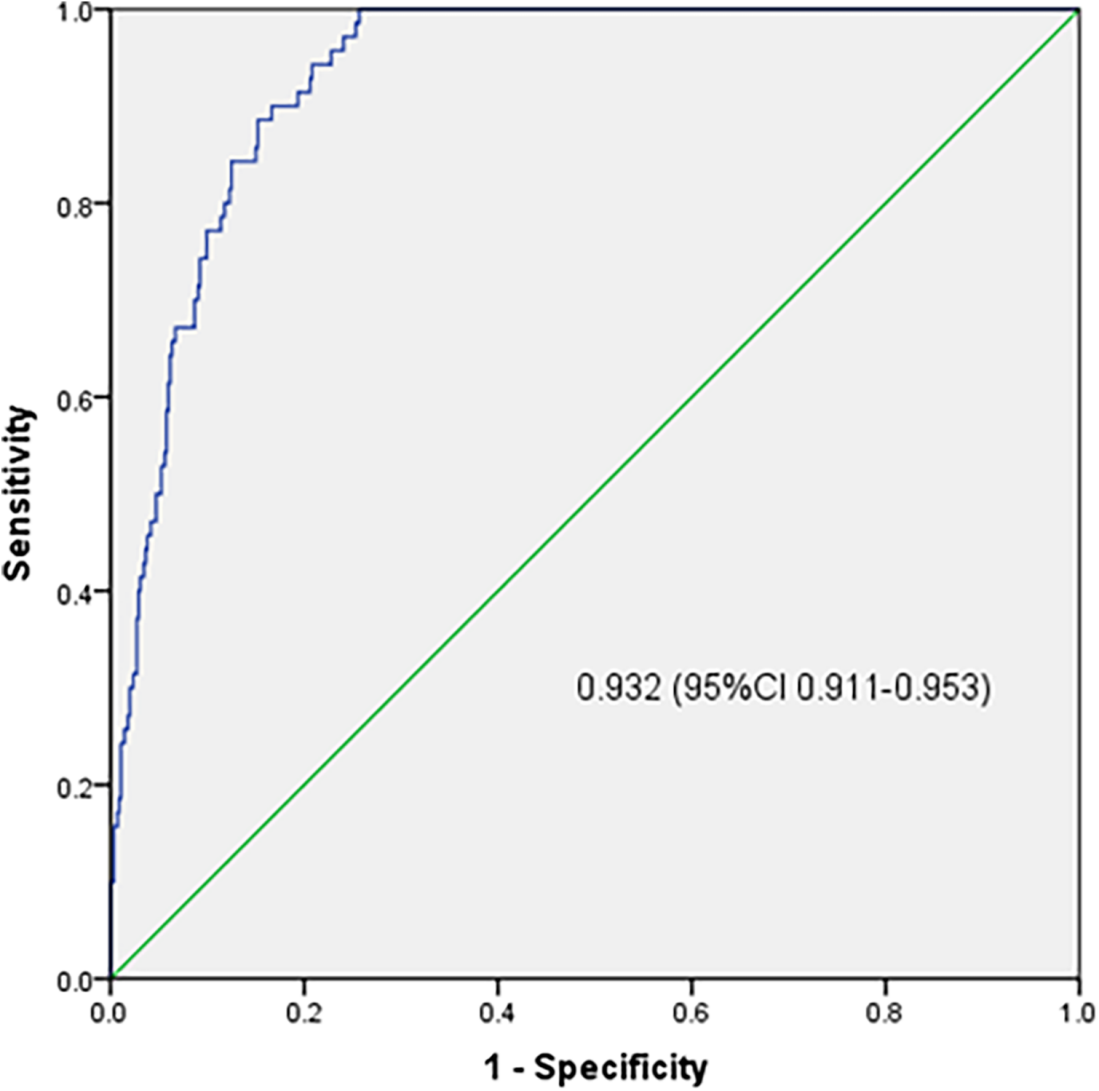
Receiver operating characteristic curve depicting performance of the developed random survival forest predictive model.

Receiver operating characteristic (ROC) curve analysis was applied to assess the performance of this RSF predictive model in the training set. Based on the RSF scores, the area under the ROC curve (AUROC) was 0.932 (95% CI, 0.911–0.953), with a sensitivity of 100%, a specificity of 74.3%, and an optimal cutoff value of 2.84 in the training set (Figure 5).

**FIGURE 6:**
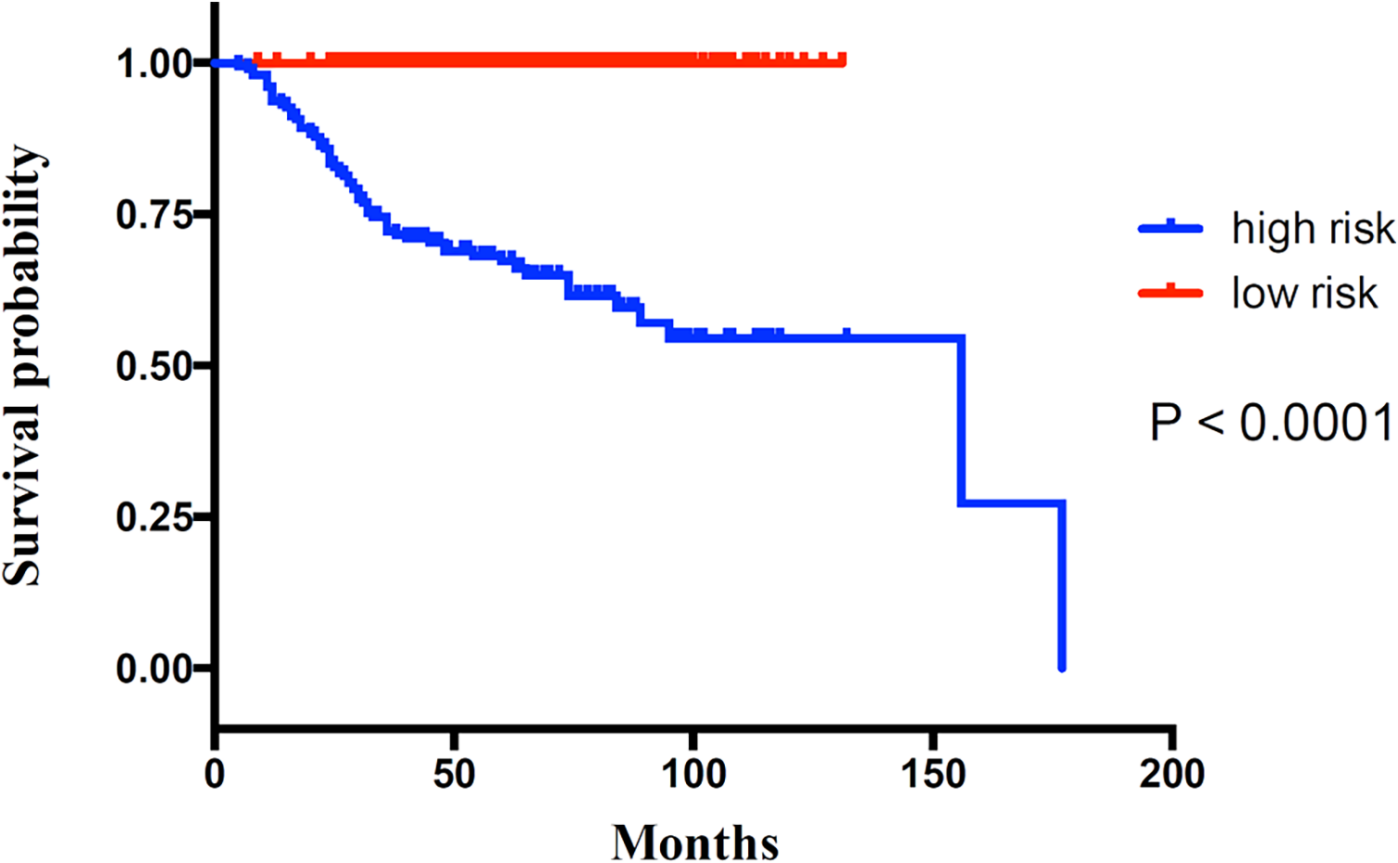
Kaplan–Meier curves for metastasis‐free survival for the training set.

The enrolled patients were divided into a high‐risk group with RSF‐based scores above the optimal cutoff value of 2.84 and a low‐risk group with RSF‐based scores below the optimal cutoff value of 2.84. The Kaplan–Meier analyses exhibited significantly different in time to metastasis‐free survival between the high‐ and low‐risk groups indicating that patients with higher prediction scores are more vulnerable to BC metastasis (*p* < .0001) (Figure 6).

The results indicated that this RSF predictive model could accurately predict the metastasis in breast cancer patients.

**FIGURE 7:**
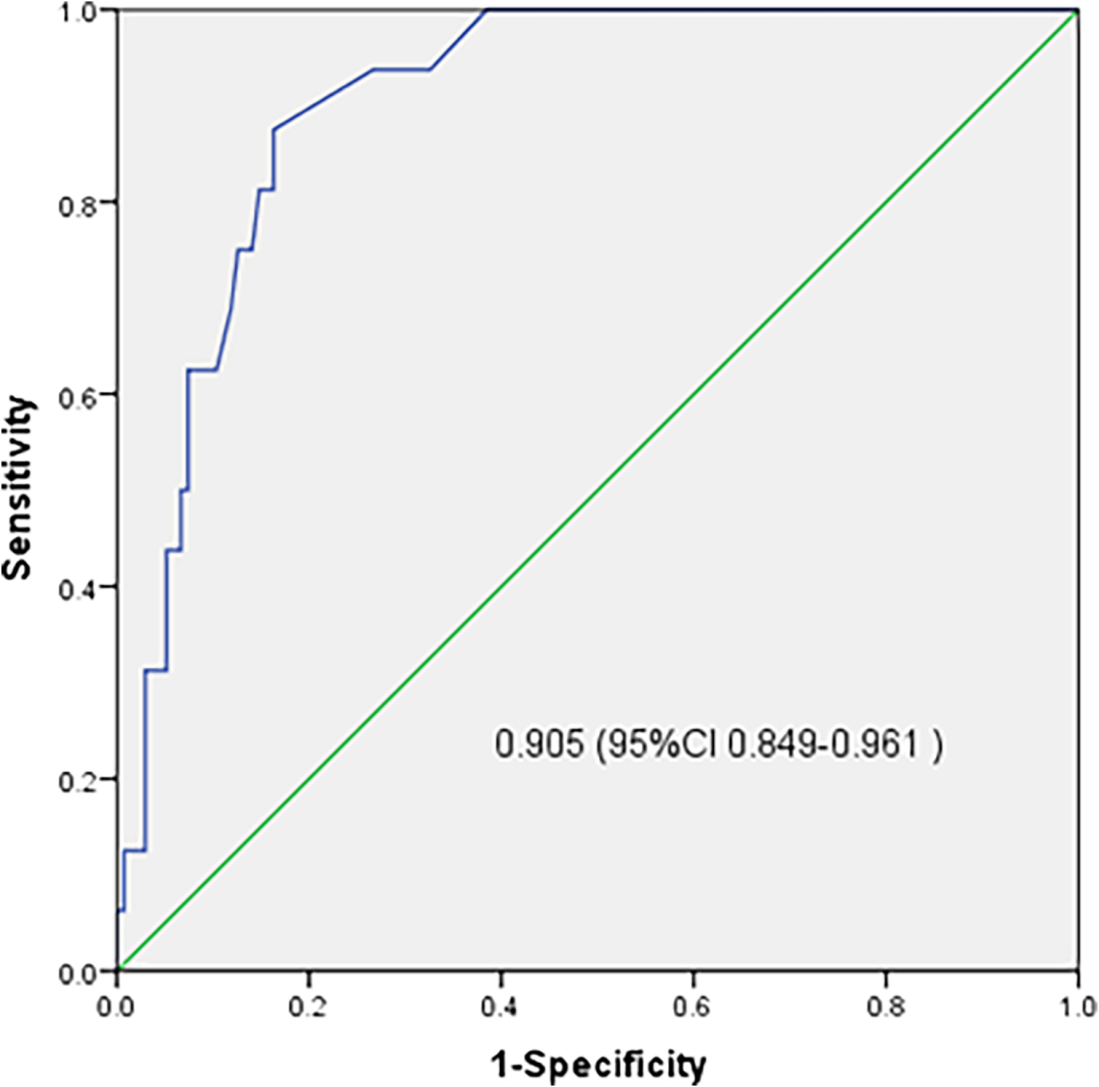
Receiver operating characteristic curve depicting performance of the developed random survival forest prognostic model in the validation set.

The predictive performance of this model was evaluated using the validation cohort. The C‐index was 0.917 (95% CI, 0.856–0.978) when the Brier score was 0.097. The AUROC achieved 0.905 (95% CI, 0.849–0.961), with a sensitivity of 100% and a specificity of 97.0% (Figure 7).

**FIGURE 8:**
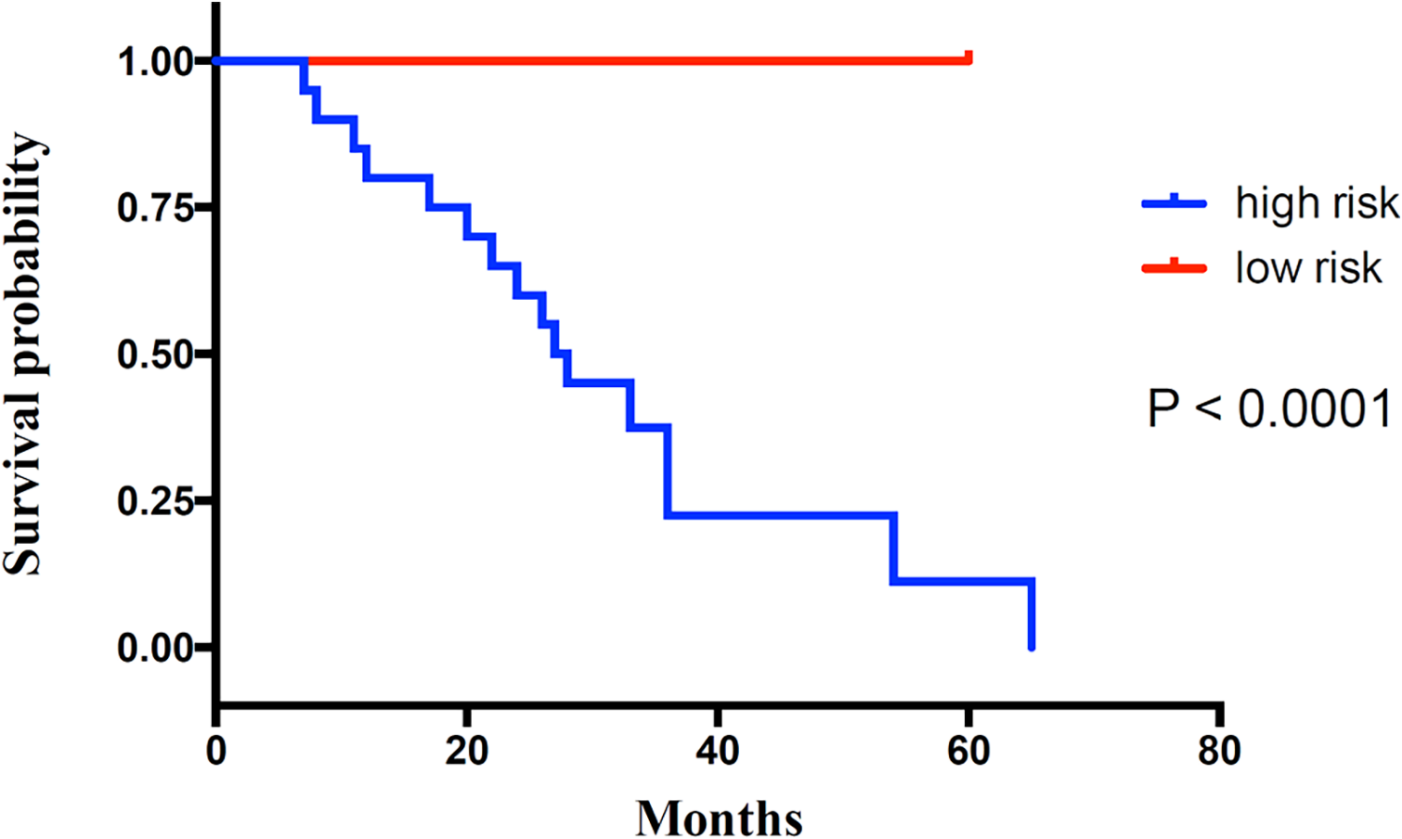
Kaplan–Meier curves for metastasis‐free survival for the validation set.

Depending on the optimal cutoff value of the RSF‐based score in the training set, patients in the validation set were divided into high‐risk group and low‐risk group. The Kaplan–Meier analyses demonstrated significantly different in time to metastasis‐free survival between the high‐risk group and low‐risk group (*p* < .001) (Figure 8) which validated the good predictive ability of this model.

## 4 DISCUSSION

The RF‐RFE algorithm,^31^ a machine learning method, was applied to automatically select the most important predictive variables to further RSF model building. Variable selection is the process of selecting a data set of predictive variables for further analysis to minimize possible generalization error.

The three best variables for this model included TNM stage, AST level, and neutrophil count. These were all from blood tests and pathological examinations, and no variable based on general information of patients was selected. However, previous studies have reported that TNM stage and neutrophil count were closely related to the prognosis of BC, and we used them to build a reliable model. The most important variable was TNM stage, which is applied widely in clinical practice to predict survival and prognosis and guide clinical decision‐making.^32^ The enzyme AST is abundantly present in hepatocytes and skeletal, cardiac, and smooth muscles and is released into the bloodstream in hepatitis, myocardial infarction, or myositis. High AST levels are independently associated with the prognosis of both hepatic tumor metastases and metastases from a primary hepatic source.^33^, ^34^ Previous studies have indicated that high AST levels may be associated with aggressive tumor biology or could be explained as a more aggressive tumor caused by high tumor cell turnover and tissue damage.^35^, ^36^ To our knowledge, this was the first study to use AST level in predicting BC metastasis. The neutrophil count is a routine blood test in clinical practice. It was reported that the change in white blood cell (WBC) count in the peripheral blood is associated with systemic inflammatory response.^37^ Further, the tumor‐related systemic inflammatory response has been proven to be an independent predictor of tumor prognosis.^38^, ^39^ The neutrophil count can reliably reflect the inflammatory status of the body, and the classification of WBC count in the peripheral blood can be used to predict BC prognosis.^37^, ^40^, ^41^, ^42^

This predictive model was established employing both patient and tumor characteristics. This predictive model had good performance in the field of validity and reliability even under external validation on an independent cohort. For the training and external validation cohorts, the C‐indexes achieved 0.959 and 0.917, respectively, showing good discrimination. The C‐indexes of previous developed predictive models ranged from 0.65 to 0.71,^15^, ^43^ which means that this model was more accurate when compared to previous models. Kaplan–Meier analyses were applied to assess the performance of this model and results indicated that our model had a good performance in predicting BC metastasis (*p* < .0001 in both the training and validation sets). Moreover, the AUROCs were 0.932 and 0.905 in the training and validation sets, respectively, which means that this model had a good predictive effect on BC metastasis. Furthermore, the AUROCs of previous developed predictive models ranged from 0.58 to 0.90, which means that this model was more accurate when compared to previous models.^44^, ^45^, ^46^ Furthermore, this model had good Brier scores of 0.113 and 0.097 for the training and external validation sets, respectively, showing good calibration.

This model based on routine demographic and clinical examination data in real‐time clinical practice and exhibits a high accuracy of prediction without increasing the medical expense and that was different from developed predictive models relied on new molecular biomarkers derived from gene or protein expression analysis. Considering that new molecular biomarkers are not tested routinely in clinical practice, the medical expense of identifying and exploying routine laboratory parameters is lower than that of employing new molecular biomarkers. Thus, this leads to additional patient expenditures and is not covered by insurance. We do not mean to negate the possible benefits of personalized care based on novel biomarkers but not all breast cancer patients can undergo the test of a novel biomarker and not all regions can perform the test for a novel biomarker. Thus, we need a practically simple and economically viable model to predict BC metastasis. The model established and verified in our study incorporated a comprehensive selected feature for both patient‐related features and tumor features to provide an easy‐to‐operate and individualized prediction of metastasis in BC patients without additional cost. This model can help clinicians stratify cases into the high and low risk of early‐stage metastasis. Thus, BC patients at low risk of metastasis can avoid the need for toxic and costly therapies, while BC patients at high risk of metastasis can undergo a more aggressive system therapy such as a more intense chemotherapy or aggressive targeted therapy for every Her‐2 positive patient and a more intense follow‐up scheme. Moreover, prediction of BC metastasis risks may better manage patient and caregiver expectations, help patients decide which therapies to choose, and even improve the patient compliance and patient care. Besides, considering life expectancy and competing risks of mortality, there is a risk of overtreatment of breast cancer in older individuals.^47^, ^48^ Predicted survival benefits, disease progress risk, effect on anticancer therapy toxicity, life expectancy, quality of life, and patient preferences should be considered carefully when making decision for older BC patients. The treatment decision making of breast cancer in older individuals should involve geriatric assessment and survival estimates.^47^, ^48^, ^49^, ^50^ Our model can provide the BC metastasis risk to contribute to make decision in the therapy of older BC patients. Over all, our model can help clinicians to provide more targeted and more accurate individualized therapy to improve the prognosis of breast cancer patients.

There were some limitations in this study. First, all enrolled patients were Han descent. Thus, this study lacks validation for other races. Therefore, validation of these results in other regions and races is needed in the future. Second, this was a retrospective cross‐sectional study performed at two centers, and the number of enrolled patients was small. In the future, studies conducted at multiple centers with larger cohorts and longer observation periods are required. Third, AST level and neutrophil count can be affected by a diverse range of factors. Our enrolled patients did not have a high elevated AST level (>5 ULN) and abnormal neutrophil count; hence, further studies are needed to verify whether this model is valid for patients with significantly abnormal AST level and neutrophil count. Fourth, not all of the breast cancer patients enrolled underwent the detection of novel biomarker. Hence, we only compared the C‐index and AUROCs between our model and other model, but there was no direct comparison of our data‐set to other models.

## 5 CONCLUSIONS

This study developed and validated a model to predict metastasis in BC patients from China. The predictive parameters were selected from routine used data in real‐time clinical practice without adding medical expense. This machine learning method based model can predict metastasis in BC patients accurately with good discrimination and calibration. Clinicians can provide precise and efficient individualized therapy for patients with BC by using this model so as to improve the prognosis of breast cancer.

## AUTHOR CONTRIBUTIONS

**Huan Li:** Conceptualization (equal); funding acquisition (equal); investigation (equal); methodology (equal); supervision (equal); validation (equal). **Ren‐Bin Liu:** Funding acquisition (equal); methodology (equal); resources (equal); validation (equal); visualization (equal); writing – review and editing (equal). **Chen‐meng Long:** Data curation (equal); formal analysis (equal); investigation (equal); project administration (equal). **Yuan Teng:** Data curation (equal); formal analysis (equal); investigation (equal). **Yu Liu:** Conceptualization (equal); formal analysis (equal); funding acquisition (equal); methodology (equal); supervision (equal); validation (equal).

## FUNDING INFORMATION

This research was supported by grants from the National Natural Science Foundation (81372815), Guangdong Basic and Applied Basic Research Foundation (2021A1515110818), the Youth Education Grand of Sun Yat‐sen University (N2019Y08), and the Guangdong Nature Science Foundation (2014A030313193).

## CONFLICT OF INTEREST STATEMENT

The authors have stated explicitly that there are no conflicts of interest in connection with this article.

## ETHICS STATEMENT

This study was performed according to the Declaration of Helsinki. The study was approved by the ethics committee of the Third Affiliated Hospital of Sun Yat‐sen University [2023‐289‐01]. All enrolled patients provided informed consent.

## Data Availability

The datasets used and/or analyzed in this study are available from the corresponding author upon reasonable request.
